# Influence of *Wolbachia* on host gene expression in an obligatory symbiosis

**DOI:** 10.1186/1471-2180-12-S1-S7

**Published:** 2012-01-18

**Authors:** Natacha Kremer, Delphine Charif, Hélène Henri, Frédérick Gavory, Patrick Wincker, Patrick Mavingui, Fabrice Vavre

**Affiliations:** 1 Université de Lyon, F-69000, Lyon; Université Lyon 1; CNRS, UMR5558, Laboratoire de Biométrie et Biologie Evolutive, F-69622, Villeurbanne, France; 2 Department of Medical Microbiology and Immunology, University of Wisconsin, Madison, WI 53706, USA; 3 Génoscope; CEA, DSV, Institut de Génomique, F-91057, Evry, France; 4 Université de Lyon, F-69000, Lyon; Université Lyon1; CNRS, VetAgro Sup, UMR 5557, Ecologie Microbienne, F-69622, Villeurbanne, France

## Abstract

**Background:**

*Wolbachia* are intracellular bacteria known to be facultative reproductive parasites of numerous arthropod hosts. Apart from these reproductive manipulations, recent findings indicate that *Wolbachia* may also modify the host’s physiology, notably its immune function. In the parasitoid wasp, *Asobara tabida*, *Wolbachia* is necessary for oogenesis completion, and aposymbiotic females are unable to produce viable offspring. The absence of egg production is also associated with an increase in programmed cell death in the ovaries of aposymbiotic females, suggesting that a mechanism that ensures the maintenance of *Wolbachia* in the wasp could also be responsible for this dependence. In order to decipher the general mechanisms underlying host-*Wolbachia* interactions and the origin of the dependence, we developed transcriptomic approaches to compare gene expression in symbiotic and aposymbiotic individuals.

**Results:**

As no genetic data were available on *A. tabida*, we constructed several Expressed Sequence Tags (EST) libraries, and obtained 12,551 unigenes from this species. Gene expression was compared between symbiotic and aposymbiotic ovaries through *in silico* analysis and *in vitro* subtraction (SSH). As pleiotropic functions involved in immunity and development could play a major role in the establishment of dependence, the expression of genes involved in oogenesis, programmed cell death (PCD) and immunity (broad sense) was analyzed by quantitative RT-PCR. We showed that *Wolbachia* might interfere with these numerous biological processes, in particular some related to oxidative stress regulation. We also showed that *Wolbachia* may interact with immune gene expression to ensure its persistence within the host.

**Conclusions:**

This study allowed us to constitute the first major dataset of the transcriptome of *A. tabida*, a species that is a model system for both host/*Wolbachia* and host/parasitoid interactions. More specifically, our results highlighted that symbiont infection may interfere with numerous pivotal processes at the individual level, suggesting that the impact of *Wolbachia* should also be investigated beyond reproductive manipulations.

## Background

Symbiotic communities of eukaryotic organisms are known to influence host developmental programs [1] and also to shape immune response against pathogens [2]. Interestingly, some genes/pathways (*e.g.* programmed cell death) have a pleiotropic role in immunity and development, and could play a major role in the maintenance of a specific bacterial community. For instance, the homeobox gene *Caudal* is involved in the formation of the antero-posterior body axis of *Drosophila*, but also in the regulation of the commensal gut microbiota [3]. In the squid-vibrio association, it has recently been shown that the regulation of a peptidoglycan recognition protein (PGRP), classically involved in innate immunity, plays a role in the activation of the apoptotic process initiating the morphogenetic changes of the symbiont-harboring organ [4]. The generality of the interplay between immunity and development during symbiosis is currently unknown.

*Wolbachia* (Anaplasmataceae) is among the most abundant intracellular bacteria. It infects both arthropods and nematodes, and is known to be a master manipulator of host biology [5]. *Wolbachia* is generally a facultative reproductive parasite in arthropods, and invades the host population by inducing cytoplasmic incompatibility, male-killing, feminization or thelytokous parthenogenesis [5].

Another extended phenotype due to the presence of *Wolbachia* is observed in the parasitoid wasp *Asobara tabida* (Hymenoptera: Braconidae), in which aposymbiotic females exhibit a strong developmental defect. Surprisingly, *Wolbachia* has become necessary for egg production in this wasp, since aposymbiotic females are unable to produce viable offspring [6]. Interestingly, *A. tabida* is the only member of the genus *Asobara* to be dependent on *Wolbachia* for oogenesis, which suggests that the dependence has evolved recently, and makes it possible to study the molecular mechanisms underlying this transition. In addition, polymorphism of the ovarian phenotype is observed in natural populations after the elimination of *Wolbachia*: some aposymbiotic females do not produce eggs, whereas others produce a few eggs that die prematurely [7,8]. This polymorphism constitutes a tool to better understand the influence of these molecular mechanisms on the severity of the ovarian phenotype and on the evolution of dependence.

At a mechanistic level, cytological analysis of the ovarian phenotype has begun to shed light on the mechanisms underlying dependence in *A. tabida*. Indeed, eliminating *Wolbachia* triggers programmed cell death (PCD) in the egg chambers within the ovaries of *A. tabida* females [9]. As egg production is tightly controlled by two main apoptotic checkpoints during oogenesis [10], the deregulation of PCD in aposymbiotic wasps must result in female inability to complete oogenesis. Because PCD is frequently involved in infection processes by bacterial pathogens [11], it has been hypothesised that a mechanism underlying the maintenance of *Wolbachia* at the individual level may have given rise to the evolution of dependence through its pleiotropic role in immunity and development [12].

This hypothesis is supported by recent findings showing that consequences of *Wolbachia* infection in insects may extend far beyond the classical effect on reproduction, by impacting host physiology and immunity. *Wolbachia* could play a role as a nutritional mutualist, by influencing iron utilization by its *Drosophila* hosts [13,14]. *Wolbachia* infection has also been shown to generate oxidative stress in one *Aedes aegypti* cell line, which reacts by the over-expression of host antioxidant genes [15]. Interestingly, Reactive Oxygen Species (ROS) are known to play a major role in immunity as a first line of defence [16] but also as a mechanism insuring microbe homeostasis [17]. Finally, *Wolbachia* is known to confer resistance against RNA viral infection in *D. melanogaster* and *D. simulans *[18,19], and against various pathogens in the mosquito *A. aegypti*, notably by priming the innate immune system [20,21].

To summarize, increasing evidence is emerging on the phenotypic effects of *Wolbachia* infection on host physiology and immunity [18,19,22]. However, few studies have attempted to describe the molecular mechanisms underlying these phenotypic effects in natural systems [20,21,23,24]. The objective of this paper is to clarify the effect of *Wolbachia* on gene expression in a particular symbiotic association in which *Wolbachia* affects developmental processes, through its effect on wasp oogenesis. For that purpose, we used both global and dedicated transcriptomic approaches.

Even though *A. tabida* is a model system in host/parasitoid and host/*Wolbachia* interactions, no genetic data were available for this parasitoid wasp. Thus, the first aim of this study was to build a reference transcriptome based on several tissues (ovaries, whole females) and physiological conditions (symbiosis, immune challenge). By sequencing 10 cDNA libraries (one of which is a normalized library), we provide here the first large-scale, genetic information on this wasp. The second aim of the study was to better understand how dependence arose in this particular species by deciphering the molecular mechanisms underlying this evolutionary transition. An overview of functions that could be differentially expressed in response to symbiosis was outlined through *in silico* analyses on ovaries EST libraries (Gene Ontology-based bioinformatics) and *in vitro* subtractions (Suppressive Subtraction Hybridizations). Then, we focused on candidate genes involved in immunity (broad sense), programmed cell death and oogenesis; functions which could play a major role in the control of ovarian phenotype through pleiotropy. Using quantitative real-time PCR, we thus characterized the effect of symbiosis on host gene expression in both males and females, in two populations exhibiting extreme ovarian phenotypes.

## Methods

### Biological system

#### Ecology

*Asobara tabida* (Hymenoptera: Braconidae) is a solitary endoparasitoid laying its eggs into the first or second instar larvae of *Drosophila* species. After *Drosophila* pupation, the parasitoid becomes an ectoparasite, and consumes its host before it itself pupates prior to emerging.

*A. tabida* is naturally infected by three strains of the intracellular bacterium *Wolbachia* (*w*Atab1, *w*Atab2 and *w*Atab3): *w*Atab1 and *w*Atab2 induce cytoplasmic incompatibility, and only *w*Atab3 is required for oogenesis completion [6,25].

#### Polymorphism of ovarian phenotype in populations

After *Wolbachia* removal, the ovarian phenotype displays a high level of intra-species variation: whereas uninfected females of the Pi strain (Pierrefeu, France) produce no eggs, uninfected females of the NA strain (Saanich, Canada) produce a small number of aborting eggs [7]. In this study, we used the NA strain and a Pi-derived strain (Pi3). Pi3 was obtained by moderate antibiotic treatment, and contains only the obligatory *Wolbachia* strain *w*Atab3 [25]. The lines are stable, and have been maintained by regular sib-matings without antibiotic treatment for about 100 generations. When comparing NA and Pi3 aposymbiotic individuals, three things must be kept in mind. (i) Any differences observed may be explained by the host genotype, whether they are directly linked to the ovarian phenotype or not. (ii) Because NA is triply infected whereas Pi3 is singly infected, differences could also be due to the presence or absence of *w*Atab1 and *w*Atab2. (iii) NA and Pi3 symbiotic individuals have differing bacterial community compositions due to the moderate antibiotic treatment of Pi3 [26].

### General procedures

#### Rearing

Wasps were allowed to parasite *Wolbachia*-free *D. melanogaster.* Insects were reared on axenic medium [27] and maintained under controlled conditions (climate chambers at 21°C, 70% relative humidity and cycle LD 12:12). Young adults (0-1 day old) were collected and anesthetized on ice before being dissected in a drop of PBS and/or stored until use at -80°C.

#### Antibiotic treatment

Because we were interested in determining the effect of symbiosis, we performed antibiotic treatments to produce *Wolbachia*-free (*i.e.* aposymbiotic) wasps. Even though antibiotics could also affect host gene expression directly (*e.g.* cytotoxicity, modification of mitochondrial metabolism) or indirectly (*e.g.* change in gut microflora), antibiotic treatment is the only efficient method to eliminate *Wolbachia* from *A. tabida*. Aposymbiotic females are sterile, and so it is impossible to establish and maintain aposymbiotic lines. Hence, antibiotic treatments had to be administered just before the experiment to obtain aposymbiotic wasps, as described in [6]. Briefly, rifampicin 2% (Hoechst, Germany) was added to the axenic nutritive medium to reach a final concentration of 2 mg/g of standard diet. Seventy *D. melanogaster* eggs were deposited in this medium, and allowed to be parasitized by three female wasps. The developing *Drosophila* thus transferred the antibiotic to each of the endoparasitoid wasp larvae, rendering them aposymbiotic. As a control, the same procedure was performed without the antibiotic treatment.

#### Bacterial challenge

Because we were interested in identifying immunity-related genes, we performed a challenge by the intracellular bacteria *Salmonella typhimurium* (strain 12023G, Grenoble) to enhance the immune response of *A. tabida* (Pi3 strain). Bacteria were prepared from a 2 h-culture initially started with a 1/10 dilution of an overnight culture (LB + ampicillin, 37°C, 190 rpm). Bacteria were rinsed twice and concentrated in 1 mL of fresh LB medium. Immune challenge was performed by injecting 13.2 nL of the mother solution (corresponding to 1.8x10^5^ bacteria) in the thorax of young (0-1 day old) females (Nanoject II injector, Drummond, Broomall, PA). As a control, 13.2 nL of fresh LB medium was injected as described above. Individuals were collected 3h, 6h and 12h after challenge (or LB injection), and stored until use at -80°C.

### Constitution of a reference transcriptome and comparison of gene expression profiles between EST libraries

#### Preparation of a normalized library for cDNA sequences acquisition

In order to build a transcriptome of reference for *A.tabida*, we constructed a normalized library (N) based on both whole females (mix of complex tissues) and ovaries (organ of interest), in various physiological conditions (with or without symbionts/pathogens). To limit host genetic variability, only the Pi3 strain was used for the library preparation. The normalized library was constructed by Evrogen (Moscow, Russia) from an equimolar proportion of total RNA prepared from aposymbiotic ovaries, symbiotic ovaries, and 3h-, 6h-, 12h-challenged symbiotic females. Total RNA samples were used for ds cDNA synthesis using the SMART approach [28]. SMART-prepared, amplified cDNA was then normalized using the DSN normalization method [29]. Normalization included cDNA denaturing/re-association, treatment by duplex-specific nuclease (DSN) [30] and amplification of normalized fraction by PCR. Normalized cDNA was purified using QIAquick PCR Purification Kit (Qiagen, Alameda, CA), digested with restriction enzyme Sfi1, purified (BD Chroma Spin - 1000 column), and ligated into pAL 17.3 vector (Evrogen) for *Escherichia coli* transformation.

#### Preparation of EST libraries for *in silico* comparisons between symbiotic and aposymbiotic ovaries

In order to increase the number of transcripts from the ovaries and to determine the influence of symbiosis on host gene expression, we constructed EST libraries on aposymbiotic (OA1 and OA2, the quality of the OA2 library being slightly lower) and symbiotic (OS) ovaries (Pi strain). Total RNA was extracted from a large number of ovaries (n_OA_=196, n_OS_=120) as described in [31], and treated with DNAse (TurboDNase, Ambion, Applied Biosystems, Austin, TX), following the Manufacturer’s instructions. Tissue libraries were prepared using Creator SMART cDNA Library Construction kit (Clontech/BD biosciences, PaloAlto, CA), following the Manufacturer’s instructions. cDNA was digested by Sfi1, purified (BD Chroma Spin – 400 column), and ligated into pDNRlib vector for *E. coli* transformation.

#### Preparation of Suppression Subtractive Hybridizations (SSH) libraries for *in vitro* comparisons

Because *in silico* comparisons of EST libraries can be limited by the depth coverage, we also used a complementary technique to compare gene expression by directly screening differentially-expressed transcripts through SSH.

In order to better understand the influence of ovarian phenotype, we performed SSHs between aposymbiotic (A) and symbiotic (S) ovaries in two populations exhibiting extreme phenotypes (Pi3: no eggs in aposymbiotic ovaries, NA: few abnormal eggs in aposymbiotic ovaries). Total RNA was extracted from a large number of ovaries [n_A_=373 and n_S_=458 for SSHs-1 A-S (Pi strain, distal part of ovaries), n_A_=n_S_=200 for SSHs-2 A-S (NA strain, whole ovaries)] and treated with DNAse (TurboDNase, Ambion, Applied Biosystems, Austin, TX), following the Manufacturer’s instructions. Amplified ds cDNA was prepared using a SMART approach [28]. SMART Oligo II oligonucleotide (Clontech/BD biosciences, PaloAlto, CA) and CDS primer were used for first-strand cDNA synthesis. SMART-amplified cDNA samples were further digested by RsaI endonuclease. Subtractive hybridizations were performed using the SSH method in both directions (Aposymbiotic *vs.* Symbiotic A/S and *vice-versa* S/A) as described in [32,33] using the PCR-Select cDNA Subtraction Kit (Clontech/BD biosciences, PaloAlto, CA). In order to reduce the number of false-positive clones in the SSH-generated libraries, the MOS procedure (Mirror Orientation Selection) was performed by Evrogen (Moscow, Russia) for SSH2s A-S, as described in [34]. Purified subtracted cDNAs from SSH1s A-S were cloned into the PCR 2.1 TOPO vector (Invitrogen, Cergy-Pontoise, France) and used for *E.coli* transformation. 137 and 72 clones (SSH1-A/S and SSH1-S/A), respectively, were selected for further confirmation. Purified cDNA from SSH2s A-S were cloned into the pAL16 vector (Evrogen) and used for *E. coli* transformation. 480 clones for each subtraction were selected for further confirmation. PCR-amplified inserts from clones representing differentially-expressed gene products were confirmed by differential hybridization using either DIG-labeled (SSH1s A-S; DIG high prime DNA labeling and detection starter kit, Roche, Meylan, France) or P-32-labeled (SSH2s A-S), subtracted cDNA probes.

Finally, in order to characterize genes responding to bacterial challenge, we performed SSHs between extracts from whole females, challenged or not challenged by *S. typhimurium* (SSHs C-NC, n_C_=n_NC_=40 females), see above for bacterial challenge procedure*.* The preparation of these SSHs has been performed by Evrogen (Moscow, Russia) with the same procedure as for SSH2s A-S.

#### EST sequencing, data processing and analysis

**Figure 1:**
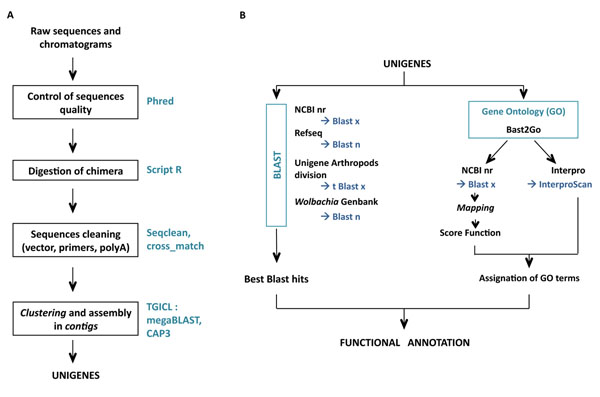
Sequence treatment (A) and functional annotation procedure (B).

All clones from the libraries were sequenced using the Sanger method (Genoscope, Evry, France), and have been deposited in the Genbank database (Normalized library: FQ829929 to FQ844492; OS: FQ848737 to FQ857191; OA1: FQ844493 to FQ848736; OA2: FQ790408 to FQ793875 and FQ859091 to FQ859175; SSH2-C: FQ828348 to FQ829118; SSH2-NC: FQ829119 to FQ829928; SSH2-A: JK217526 to JK217700 and JK217743 to JK217748; SSH2-S: JK217375 to JK217525 and JK217729 to JK217742; SSH1-S: JK217749 to JK217767; SSH1-A: JK217701 to JK217728). A general overview of the Expressed Sequence Tags (ESTs) data processing is given in Figure 1. Raw sequences and traces files were processed with Phred software [35,36] in order to eliminate any low quality bases in sequences (score < 20). Sequence trimming, which includes polyA tails/vector/adapter removal, was performed by Cross_match. Chimeric sequences were computationally digested into independent ESTs.

Clustering and assembly of the ESTs were performed with TGICL [37] to obtain putative unique transcripts (unigenes) composed of contiguous ESTs (contigs) and unique ESTs (singletons). To do this, a pairwise comparison was first performed using a modified version of megablast (minimum similarity 94%). Clustering was done with tclust, which proceeds by a transitive approach (minimum overlap: 60 bp at 20 bp maximum of the end of the sequence). Assembly was done with CAP3 (minimum similarity 94%).

To detect unigene similarities with other species, several blasts (with high cut-off e-values) were performed against the following databases: NCBI nr (blastx (release: 1 March 2011); e-value < 5, HSP length > 33aa), Refseq genomic database (blastn, e-value < 10), Unigene division Arthropods (tblastx, #8 *Aedes aegypti*, #37 *Anopheles gambiae*, #3 *Apis mellifera*, #3 *Bombyx mori*, #53 *Drosophila melanogaster*, #9 *Tribolium castaneum*; e-value < 5), *Nasonia vitripennis* Nvit OGS_v1.0 (CDS predicted by Gnomon (NCBI)) and *Wolbachia* sequences from Genbank (blastn (release 164); e-value < e-20). Gene Ontology annotation was carried out using Blast2go software [38]. During the first step (mapping), a pool of candidate GO terms was obtained for each unigene by retrieving GO terms associated with the hits obtained after a blastx search against NCBI nr. During the second step (annotation), reliable GO terms were selected from the pool of candidate GO terms by applying the Score Function (SF) of Blast2go with permissive annotation parameters (EC_weight=1, e-value_filter=0.1, GO_weight=5, HSP/hit coverage cut-off=0%). In the third step of the annotation procedure, the pool of GO terms selected during the annotation step was merged with GO terms associated with Interpro domain (Interpro predictions based on the longest ORF). Finally, the Annex augmentation step was run to modulate the annotation by adding GO terms derived from implicit relationships between GO terms [39].

In order to extract the biological processes and molecular functions statistically over-represented in aposymbiotic libraries, we performed a hyper-geometrical test between GO terms from the aposymbiotic libraries (OA1 and OA2) and those from the OS library, which corresponds to natural physiological conditions. The p-values were then adjusted using Bonferroni’s correction. To perform a functional enrichment analysis of the unigenes extracted from the SSH, we used the FatiGO web tool [40] on the OS library. With respect to the GO analysis, levels 3 and 6 were chosen to describe biological processes, and level 4 was chosen to describe molecular functions.

### Gene expression measurement by quantitative RT-PCR (qRT-PCR)

We sought to determine the effect of symbiosis on the expression of a set of candidate genes involved in immunity, programmed cell death and oogenesis. For that purpose, we first compared gene expression between symbiotic and aposymbiotic samples, in ovaries (to characterize the dependence phenotype induced by *Wolbachia*) and then in males (to provide additional information concerning the specificity of the process). In order to limit the influence of the presence of eggs in symbiotic *vs.* aposymbiotic ovaries of the Pi3 strain, only the distal part that does not contain eggs (DPOv) was dissected in that strain. Because the dependence phenotype is determined by the host genotype [8], we compared gene expression between two populations exhibiting extreme ovarian phenotypes.

Total RNA was extracted from 5 replicates of 10 males or 10 full (NA)/partial (Pi) ovaries, as described in [31]. Total RNA was purified from potential DNA contamination by DNase treatment (Turbo DNAse, Ambion, Applied Biosystems, Austin, TX). First-strand cDNA synthesis was performed from 500 ng of total RNA using the Superscript III enzyme (Invitrogen, Cergy-Pontoise, France) and oligodT primers, according to the Manufacturer’s instructions. For each biological sample, 4 ng of cDNA was spotted in duplicate in a 96-well plate (Microlab star, Hamilton, Bonaduz, Switzerland). Quantitative PCR was performed using LightCycler LC480 system (Roche, Meylan, France) as follows: 5 min at 95°C, 35 times [15 s at 95°C, 10s at 58°C, 20 s at 72°C], 20 s at 70°C. A melting curve was recorded at the end of the PCR amplification to confirm that a unique transcript product had been amplified. The reaction mixture consisted of 0.5 µM of each primer, 5 µL of Fast SYBR-Green Master Mix (Roche, Meylan, France), and 4 µL of diluted cDNA (corresponding to 4 ng of cDNA). Primers used for quantitative PCR are summarized in Additional File 1. In order to calculate PCR efficiencies, standard curves were plotted using seven dilutions (10–10^7^ copies) of a previously amplified PCR product purified using Nucleospin Extract II kit (Macherey-Nagel, Hoerdt, France). Expression data were estimated by calculating E^−Cp^, where E corresponds to the efficiency of the PCR reaction, and Cp to the crossing point [41]. Candidate gene expression was normalized by the geometric mean of the expression level of three housekeeping genes (Ribosomal L6, β-tubulin, and Elongation factor 1γ), and analyzed by Wilcoxon’s test. The p-values were then adjusted using false discovery rate’s correction (FDR, R software, version 2.12).

## Results

### More than 12,000 unigenes sequenced in cDNA libraries

**Figure 2:**
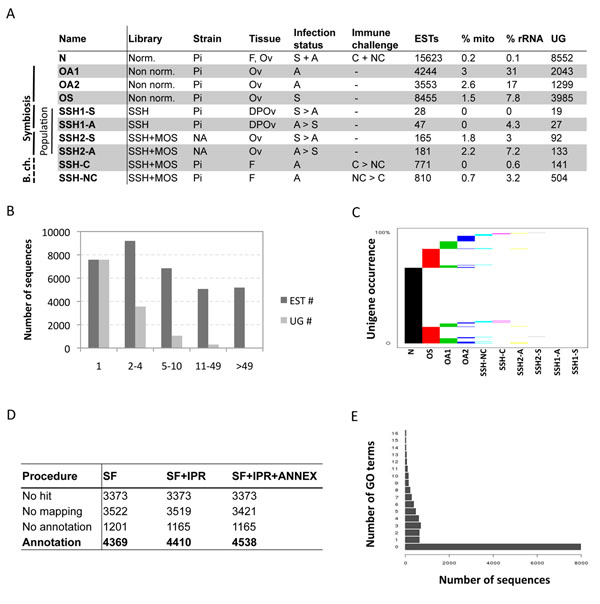
**Characteristics of the EST libraries A.** Summary of the different EST libraries from *Asobara tabida*, used to build a transcriptomic map, but also to address the question of the effect of symbiosis and bacterial challenge (b. ch.) on host gene expression. cDNA libraries were sequenced with or without normalization (Norm. or Non norm., respectively). Suppression Subtractive Hybridizations (SSHs) were performed with or without the Mirror Orientation Selection procedure (MOS). The influence of ovarian phenotype was addressed using two different populations known to exhibit extreme phenotypes after *Wolbachia* removal: females from the Pi3 strain (Pierrefeu, France) do not produce any eggs, while females from the NA strain (Saanich, Canada) produce a few eggs that fail to develop normally. Immune challenge was performed by injecting 1.8x10^5 ^*Salmonella typhimurium* in aposymbiotic females, and RNA was extracted 3h, 6h and 12h after challenge. Abbreviations stand for: DPOv: Distal Part of the Ovaries (*e.g.* without the eggs), Ov: Ovaries, F: Females, S: Symbiotic, A: Aposymbiotic, C: immune Challenge, NC: No immune Challenge. ESTs: Expressed Sequenced Tags, mito: mitochondrial genes, rRNA: ribosomal RNA, UG: number of unigenes found after a clustering/assembly. **B.** Abundance classes of ESTs and Unigenes. **C.** Unigene occurrences among the EST libraries. The horizontal axis represents the different EST libraries. The occurrence of unigenes within the libraries is shown on the vertical axis. A horizontal reading of the graph indicates the percentage of unigenes shared by several EST libraries. **D.** Gene Ontology (GO) annotation results for High Scoring Pair (HSP) coverage of 0%. GO annotation was first carried out using the Score Function (SF) of the Blast2go software. The GO terms selected by the annotation step were then merged with Interproscan predictions (SF+IPR). Finally, the annex augmentation was run (SF+IPR+ANNEX). **E.** Annotation distribution of GO terms.

To construct a major dataset on the transcriptome of *A. tabida*, ESTs were generated from several strains and tissues of wasps with different *Wolbachia*-infection and immune-challenge status. The different combinations represent a total of 10 cDNA libraries, including 6 Subtractive Suppression Hybridization (SSH) libraries, 3 non-normalized libraries, and one normalized library. Characteristics of these cDNA libraries are summarized in Figure 2A. In brief, a total of 33,877 ESTs were generated using the Sanger sequencing approach. The average length of these sequences after cleaning was 522 ± 160 bp. EST assembly was done by TGICL [37] on all EST sequences, leading to 12,511 unique transcripts (*i.e.* unigenes) composed of contiguous ESTs (*i.e.* contigs) or unique ESTs (*i.e.* singletons). The average length of these unigenes was 657 ± 300 bp, for an average depth of 3.8 (see abundance classes in Fig. 2B). The average GC content was 39.5%. Sequences covered around 8.2 Mb *vs.* 33 Mb of predicted transcripts in *Nasonia vitripenis*, and 14 Mb in *Drosophila*. Consequently, this first sequencing data set gives reliable information about the transcriptome of *A. tabida*.

However, most unigenes were obtained from the normalized library and the ovary libraries (Fig. 2C). In addition, the overlap between libraries was low, suggesting that the sampling effort should be increased to perform a transcriptomic analysis at the gene level. Indeed, 60% of the unigenes were defined by a single EST (Fig. 2B). Furthermore, the two aposymbiotic libraries (OA1 and OA2) only partially overlapped (Fig. 2C), sharing 345 unigenes, corresponding to 16% of OA1 and 26% of OA2, respectively.

Functional annotation was performed on the 12,511 unigenes using Blast against various databases and using the Gene Ontology procedure (method summarized in Fig. 1B, results in Fig. 2D). Gene Ontology (GO) is a structured vocabulary describing gene products with terms taken from different ontologies, such as the molecular function or biological process resulting from the coordinate action of molecular functions. We chose high e-value cut-offs because of the ancient divergence between *A. tabida* and the closest sequenced genomes. In addition, divergence can be very high for fast-evolving genes like immune effectors. The principal database sources for the GO annotation were UniprotKB (55%), Flybase (21%) and Mouse Genome Informatics (19%). Around 70% of the unigenes had Blast similarities, mainly against *N. vitripennis* (15 %), *Apis mellifera* (13%), *Harpegnathos saltator* (11%), *Camponotus floridanus* (11%), *Solenopsis invicta* (8%) and *Tribolium castaneum* (2%), with an e-value lower than e^-20^ for more than 55% of the unigenes. Undetectable similarity could correspond to the UTR part of the cDNA, or to species-specific genes. Around 40% of unigenes were annotated after the Blast2go annotation procedure for High Scoring Pair (HSP) over a hit length coverage cut-off of 0%. We used permissive annotation parameters since our goal was to keep the maximum functional annotation even if it involves only a very short portion of the unigene (*e.g.* a domain). Adding Interproscan prediction and running the Annex augmentation procedure increased the number of unigenes annotated. While we kept the unigenes/GO datatset corresponding to the minimum HSP coverage percentage, the mean number of GO terms assigned per unigene was 1.66 GO (Fig. 2E).

### Functional analysis of the symbiotic interaction

To determine the effect of *Wolbachia* on host gene expression, we first compared the libraries from aposymbiotic ovaries (OA1 and OA2) to the reference library based on symbiotic ovaries (OS), which represents the natural physiological condition of the wasp. This analysis was performed in the Pi3 strain, which exhibits a strong ovarian phenotype. In total, 5955 unigenes were present in these three libraries, 3764 of which occurred only once. The low sequencing depth made it difficult to detect significant differences at the gene level. Hence, to get a better idea of the biological functions that respond to symbiosis, we extracted all the functional annotations from the unigenes, and performed a function-based analysis (Table 1 for biological process level 3 and molecular function level 4; Additional File 2 for biological process level 6). Autophagic (level 3) and apoptotic processes (level 6) were over-represented in aposymbiotic ovaries. Developmental processes (*e.g.*, reproductive developmental process (level 3) including female gonad development (level 6)) and interspecies interactions between organisms were also over-represented in the aposymbiotic ovaries library. Interestingly, numerous molecular functions over-represented in the aposymbiotic ovaries library were linked to stress regulation (*e.g.*, chaperone binding; glutathione peroxidase activity; oxidoreductase activity linked to superoxide radicals, peroxide, heme-copper or NADH; monooxygenase activity) or immune recognition (*e.g.*, lipoprotein binding, liposaccharide binding).

**Table 1:** Functions under-represented in wasp ovaries in response to *Wolbachia* infection

	**Biological process**	**GO**	**A**	**S**	**A/S**	**1+2**
	
**OA1, level 3**	autophagy	GO:0006914	0,07	0,01	**7,00**	
(n = 95)	interspecies interaction between organisms	GO:0044419	0,12	0,02	**6,00**	
	stem cell maintenance	GO:0019827	0,05	0,02	**2,50**	*
	temperature homeostasis	GO:0001659	0,02	0,01	**2,00**	*
	mRNA splice site selection	GO:0006376	0,26	0,13	**2,00**	
	muscle attachment	GO:0016203	0,26	0,14	**1,86**	
	reproductive developmental process	GO:0003006	0,52	0,3	**1,73**	
	generation of precursor metabolites and energy	GO:0006091	3,23	2,16	**1,50**	
	biosynthetic process	GO:0009058	13,08	9,27	**1,41**	
	cellular component organization and biogenesis	GO:0016043	17,84	16,46	**1,08**	
	ensheathment of neurons	GO:0007272	0,02	0	**-**	
	transposition	GO:0032196	0,05	0	**-**	*
						
**OA2, level 3**	temperature homeostasis	GO:0001659	0,17	0,01	**17,00**	*
(n = 16)	stem cell maintenance	GO:0019827	0,06	0,02	**3,00**	*
	transposition	GO:0032196	0,03	0	**-**	*
						
						
	**Molecular function**	**GO**	**A**	**S**	**A/S**	**1+2**
	
**OA1, level 4**	chaperone binding	GO:0051087	0,12	0,02	**6,00**	*
(n =105)	glutathione peroxidase activity	GO:0004602	0,16	0,04	**4,00**	
	cell adhesion molecule binding	GO:0050839	0,14	0,04	**3,50**	
	oxidoreductase activity, acting on superoxide radicals as acceptor	GO:0016721	0,05	0,02	**2,50**	
	transferase activity, transferring alkyl or aryl (other than methyl) groups	GO:0016765	0,26	0,11	**2,36**	*
	flavine monoNucleotid binding	GO:0010181	0,09	0,04	**2,25**	
	protein transmembrane transporter activity	GO:0008320	0,68	0,33	**2,06**	
	lipoprotein binding	GO:0008034	0,02	0,01	**2,00**	*
	oxidoreductase activity, acting on peroxide as acceptor	GO:0016684	0,16	0,08	**2,00**	
	transferase activity, transferring sulfur-containing groups	GO:0016782	0,02	0,01	**2,00**	
	phosphopantetheine binding	GO:0031177	0,16	0,08	**2,00**	
	lipoic acid binding	GO:0031405	0,14	0,07	**2,00**	
	ice binding	GO:0050825	0,02	0,01	**2,00**	*
	substrate-specific transmembrane transporter activity	GO:0022891	5,07	2,89	**1,75**	
	translation elongation factor activity	GO:0003746	1,3	0,79	**1,65**	
	heme-copper terminal oxidase activity	GO:0015002	1,04	0,67	**1,55**	
	oxidoreductase activity, acting on heme group of donors	GO:0016675	1,04	0,67	**1,55**	
	oxidoreductase activity, acting on NADH or NADPH	GO:0016651	1,01	0,7	**1,44**	
	hydrolase activity, acting on acid anhydrides	GO:0016817	7,45	5,25	**1,42**	
	active transmembrane transporter activity	GO:0022804	1,37	0,98	**1,40**	
	electron carrier activity	GO:0009055	1,44	1,04	**1,38**	
	fatty acid binding	GO:0005504	0,24	0	**-**	*
	pheromone binding	GO:0005550	0,05	0	**-**	
	polysaccharide binding	GO:0030247	0,02	0	**-**	*
						
**OA2, level 4**	ice binding	GO:0050825	0,17	0,01	**17,00**	*
(n = 92)	chaperone binding	GO:0051087	0,08	0,02	**4,00**	*
	oxidoreductase activity, acting on paired donors, with incorporation or reduction of molecular oxygen	GO:0016705	0,62	0,19	**3,26**	
	lipoprotein binding	GO:0008034	0,03	0,01	**3,00**	*
	transferase activity, transferring sulfur-containing groups	GO:0016782	0,03	0,01	**3,00**	*
	thiamin pyrophosphate binding	GO:0030976	0,03	0,01	**3,00**	
	monooxygenase activity	GO:0004497	0,68	0,24	**2,83**	
	fatty acid binding	GO:0005504	0,03	0	**-**	*
	polysaccharide binding	GO:0030247	0,06	0	**-**	*
	protein self-association	GO:0043621	0,06	0	**-**	

GO terms represented differently in libraries from aposymbiotic (A) and symbiotic (S) ovaries (Pi3 strain). The proportion of ESTs related to each GO function is indicated in the OA libraries (OA1 and OA2) and in the reference library (OS). Functions are sorted relative to their A/S ratio, representing the enrichment percentage in the OA library compared to the OS library. An asterisk indicates a function over-represented in both OA1 and OA2 libraries.

Another way of detecting biological functions responding to symbiosis is to directly screen for genes that are differentially expressed after *in vitro* subtractions between cDNA libraries. We therefore performed two different Suppressive Subtraction Hybridizations (SSHs) in populations exhibiting extreme ovarian phenotypes after the removal of *Wolbachia*, in order to determine the influence of the ovarian phenotype on gene expression. The first SSH was carried out on the Pi3 strain, in which aposymbiotic females do not produce eggs; and the second was carried out on the NA strain, in which aposymbiotic females produce a few ‘abnormal’ eggs. Functions over-represented in aposymbiotic ovaries (SSH1-A and SSH2-A) relative to symbiotic ovaries (OS) were analyzed by the FatiGO web tool (Table 2). In the Pi3 strain, genes involved in ferric iron binding were over-represented in aposymbiotic ovaries, whereas those involved in cell cycle regulation and ribosomal machinery were over-represented in the NA strain. Interestingly, both *in silico* and *in vitro* subtractions between symbiotic and aposymbiotic ovaries highlighted the role of host homeostasis (especially through iron and oxidative stress regulation), and the *Ferritin* gene was over-expressed in aposymbiotic individuals in all these comparisons (data not shown).

**Table 2:** Functional enrichment analysis

Test	N	Process	Level	GO terms	GO number	p-value	adj. p-value
SSH2A *vs.* OS	127	Biological process	3	cell cycle	GO:0007049	1.2 e-4	4.4e-3
				cellular component organization & biogenesis	GO:0016043	1.0 e-4	4.4e-3
			4	ribonucleoprotein complex biogenesis & assembly	GO:002613	1.7e−5	3.1e-3
				organelle organization & biogenesis	GO:0006996	5.5e−5	4.9e-3
			5	ribosome biogenesis & assembly	GO:0042254	7.2e−6	2.6e-3
							
		Molecular function	7	structural constituent of ribosome	GO:0003735	1.1 e-4	8.8e-3
							

SSH1A *vs.* OS	26	Molecular function	7	ferric iron binding	GO:0008199	2.0e-4	4.4e-2
							

SSH2S *vs.* OS	88			no significant terms			
							

SSH1S *vs.* OS	10			no significant terms			
							

GO terms represented differently in SSH experiments comparing aposymbiotic ovaries (A) and symbiotic ovaries (S) in two different populations. SSH1 was performed in the Pi3 strain in which females do not produce eggs (tissue: distal part of the ovaries). SSH2+MOS was performed in the NA strain in which females produce a small number of ‘abnormal’ eggs (tissue: whole ovaries).

Suppressive Subtraction Hybridizations were performed between wasps challenged with *S. typhimurium* and unchallenged wasps (SSHs C-NC) in order to detect immune genes. However, the SSH-C was saturated with the antimicrobial peptide *Hymenoptaecin*, and so was not informative.

### Expression of genes related to immunity (broad sense), programmed cell death, and oogenesis

**Figure 3:**
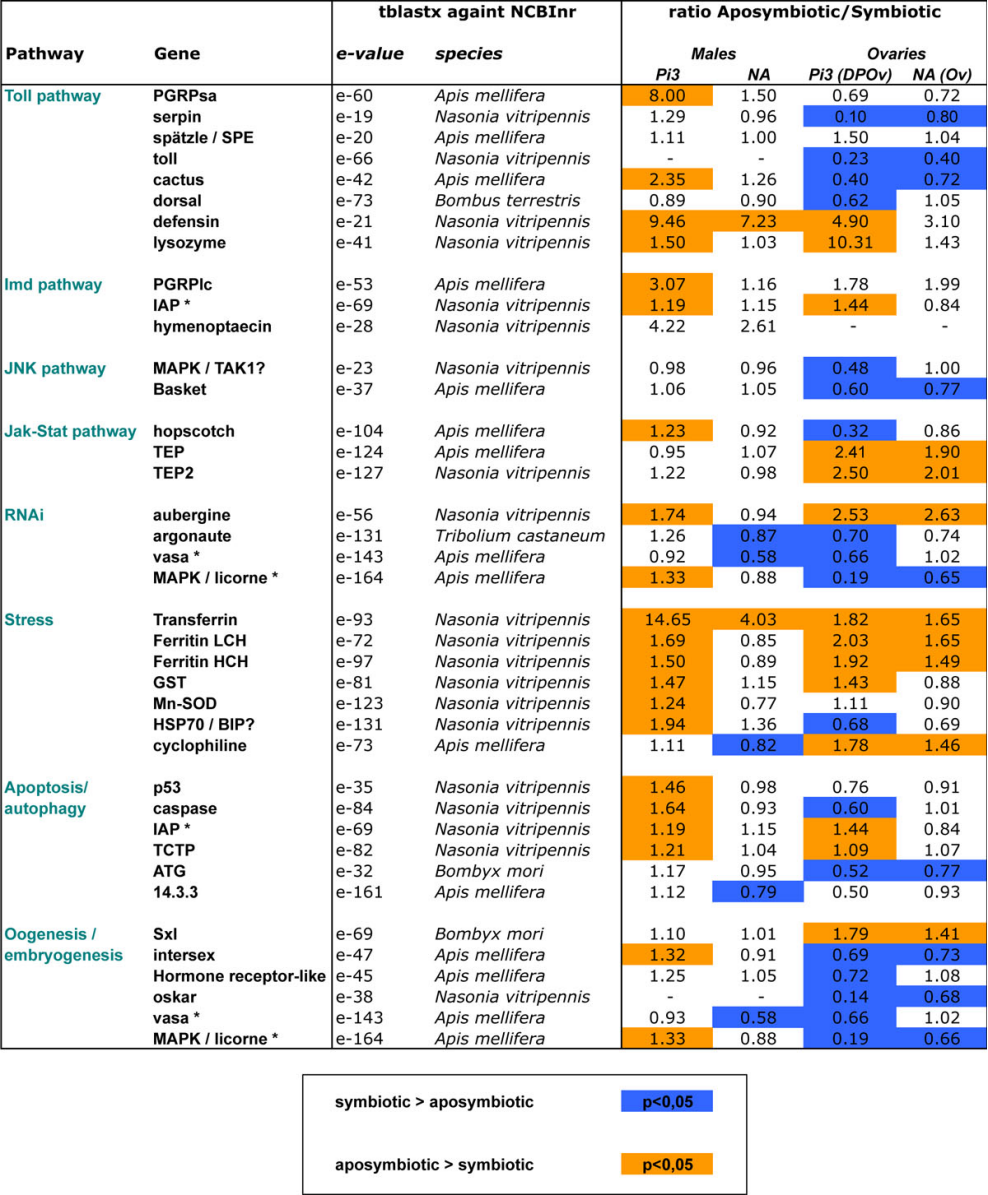
**Differential expression of candidate genes in response to *Wolbachia* infection, depending on tissue and population.** The Pi3 strain exhibits a strong ovarian phenotype after *Wolbachia* removal (no eggs in the ovaries), while the NA strain produces a few eggs that fail to develop normally. Quantitative RT-PCR was performed either in males or in ovaries (whole ovaries for the NA strain, and a distal part of the ovaries (DPOv) for the Pi3 strain). Details of the expression patterns are given in Additional file 3. The ratios between the average expression under aposymbiotic and symbiotic conditions are given. Genes over-expressed in symbiotic individuals are highlighted in blue; whereas those over-expressed in aposymbiotic individuals are highlighted in orange (Wilcoxon’s test on expression data, p-values adjusted using FDR’s correction). A dash indicates that there is no expression in the given tissue. Genes have been ordered within signaling pathways, and from the receptors to the effectors in immune pathways. Asterisks are assigned to pleiotropic genes implicated in several biological functions. PGRP: PeptidoGlycan Recognition Protein, SPE: Spätzle-Processing Enzyme, IAP: Inhibitor of APoptosis, TEP: ThiolEster-containing Protein, LCH: Light Chain, HCH: Heavy Chain, GST: Gluthatione-S-Transferase, SOD: SuperOxide Dismutase, HSP: Heat Shock Protein, TCTP: Translationally-Controlled Tumor Protein, ATG: Autophagy-related protein, Sxl: Sex-Lethal, MAPK: MAP kinase.

Previous cytological analyses had shown that the oogenetic defects due to the elimination of *Wolbachia *[6] are associated with an increase in programmed cell death (PCD) in the ovaries [9]. In addition to these findings, the global transcriptomics analysis highlighted the fact that removing *Wolbachia* might interfere with signaling pathways related to immunity in its broad sense, including stress regulation. We used our reference transcriptome to choose unigenes related to these pathways (immunity, PCD, oogenesis), and studied their expression by qRT-PCR (Fig. 3, detailed expression pattern in Additional File 3). Unfortunately, it was not possible to study all the genes in these signaling pathways. Hence, we chose those that were the most characteristic of a given pathway and the best annotated using Blast. We first studied their expression in response to *Wolbachia* removal, by comparing symbiotic and aposymbiotic samples, in both ovaries and males. Indeed, the comparison of the two tissue types can provide additional information about the specificity of the process: (i) gene expression can be observed throughout the male, in which case there is no evidence of apoptotic phenotype or (ii) expression can be specific to the ovaries, in which case an apoptotic phenotype and an oogenetic defect are detected [6,9]. In the latter case however, the response could also reflect female specificity or any degree of tissue specificity. As the ovarian phenotype is controlled by the host genotype [8], we finally compared gene expression in response to symbiosis between two different populations with contrasting ovarian phenotypes.

Overall, the expression patterns observed in males and ovaries differed considerably in terms of expression level and response to *Wolbachia* removal, highlighting either tissue-specific or sex-specific expression and response. While most genes displayed a differential response to bacterial infection under at least one condition (tissue/population combination), the difference in expression was greater than 2-fold (ratio higher than 2 or lower than 0.5) in only one in six of the comparisons, showing that the impact of *Wolbachia* removal on expression was qualitatively important, but quantitatively limited (Table 3). As expected, expression was more affected in the ovaries than in the males for both strains (Pi strain, χ^2^=9.38, p=0.009; NA strain, χ^2^=6.67, p=0.035). The fact that expression was affected to a greater extent in Pi3 than in NA ovaries was also expected (χ^2^=15.59, p=0.0004). More surprisingly, the same pattern was observed in males (χ^2^=10.77, p=0.004), although no clear phenotype has ever been identified in males. This indicates that the difference in gene expression between Pi3 and NA ovaries was not solely attributable to the ovarian phenotype.

**Table 3:** Overall analysis of differential gene expression in response to *Wolbachia* removal

	Males			Ovaries	
			
	Pi	Na		Pi	Na
**Total**	34	34		35	35
**DE**	19	6		30	16
**DE>2**	5	2		14	3
**Non DE**	15	28		5	19

Differentially-expressed (DE) genes are those of which the expression, estimated by qRT-PCR, was statistically different under aposymbiotic (A) and symbiotic (S) conditions (Wilcoxon’s test on expression data, p-values adjusted using FDR’s correction, see details in Figure 3). DE>2 corresponds to the number of DE genes with an aposymbiotic/symbiotic expression ratio that is greater than 2 or smaller than 0.5. The Pi3 strain exhibits a strong ovarian phenotype after *Wolbachia* removal (no eggs in the ovaries), while the NA strain produces a few eggs that fail to develop normally.

If we focus on genes involved in immunity (Toll, Imd, JNK, JAK-STAT, RNAi pathways), expression patterns were relatively clear in males. Indeed, NA males showed a very limited response to *Wolbachia* removal, whereas Pi3 males mainly showed an under-expression of these immune genes in symbiotic individuals. On the opposite, immune genes were mainly over-expressed in symbiotic ovaries of both strains, with however a higher differential expression in Pi3 ovaries. This difference could be attributable to the ovarian phenotype, but also to other phenotypic traits controlled by the female genotype. Furthermore, numerous genes involved in immune functions (*e.g. Toll*, *Cactus*, *Dorsal*, *Basket*) may also play an important role during the development. Since their transcripts may accumulate during oogenesis, expression results associated with these genes have to be interpreted with caution in aposymbiotic females whose oogenetic process is markedly affected. Curiously, in most of these immune pathways, but particularly the Toll and JAK-STAT pathways, expression profiles depended on the gene being investigated. Indeed, genes upstream in the pathways were mainly over-expressed in symbiotic individuals, whereas downstream effectors, such as anti-microbial peptides and *TEPs*, were mainly down-regulated in response to symbiosis. It is also interesting to note that gene expression was generally much lower in ovaries than in males, suggesting that this tissue may display limited immuno-competency.

In order to study immunity in its broad sense, we also took into account processes involved in the stress response and programmed cell death, as they can also be involved in limiting bacterial infection. Unfortunately, very few genes involved in canonical pathways of apoptosis and autophagy were detected among the libraries, which limited the scope of our investigation. Expression patterns were once again very different in NA males and Pi3 males. In Pi3 males, genes involved in stress and programmed cell death were mainly under-expressed in response to symbiosis. It is difficult to interpret the response of NA males to symbiosis, since the very few genes that were differentially regulated were either up or down-regulated within a given pathway. In the ovaries, where cytological analyses have highlighted apoptotic and autophagic processes in aposymbiotic ovaries [[9],Rancès, pers. com.], processes associated with PCD were either unchanged in response to symbiosis (NA strain) or, surprisingly, over-expressed in symbiotic ovaries (Pi3 strain). In Pi3 and NA ovaries, genes involved in the stress response (detoxification, folding) were mainly under-expressed in response to symbiosis, which confirms the trend highlighted by the analyses of EST libraries.

*Wolbachia* is known to play a role in oogenesis completion in *A. tabida *[6], and to restore fertility to the Sxl^f4 ^*D. melanogaster* mutant [42]. Therefore, we studied the expression of genes known to be involved in sex determination in *Drosophila* (*Sxl*, *Ix*) and also in oogenesis and embryogenesis. Expression of *Sxl* and *Ix* was not limited to one sex, as shown by [43], and varied in response to symbiosis in all the populations investigated. However, the function of *Sxl* in sex determination seems to be restricted to *Drosophila* and could have another role (not female specific) in other insect species [44]. Genes involved in oogenesis and embryogenesis were all over-expressed in symbiotic ovaries, and more significantly so in the Pi ovaries. These findings are thus congruent with the ovarian phenotype of aposymbiotic females (without eggs in the Pi3 strain, and with a few eggs in the NA strain). Patterns in gene expression could be explained by the ovarian phenotype’s being related either to a direct role in oogenesis or to mRNA storage in the eggs for subsequent embryo development.

## Discussion

Phenotypic effects of *Wolbachia* on host biology are being increasingly reported in arthropod species [22]. Furthermore, growing numbers of *Wolbachia* genomes have now been sequenced from strains inducing various phenotypic effects [45-49], which provides essential information about the biology and evolution of the symbiont. However, very few studies have focused on the overall response of the host to the presence of *Wolbachia* in natural associations [20,21,23,24]. Most studies have focused on host response after stable [20,21] or transient infection by *Wolbachia *[50], or in cell cultures [23,51].

The first goal of this work was to generate a first reference transcriptome of *A. tabida*, a model system both for host/*Wolbachia *[12] and host/parasitoid interactions [52,53]. The 12,511 unigenes we isolated from the wasp *A. tabida* constitute a valuable resource for further genetic studies of these interactions. For example, the host transcriptional response to parasitoid attack has been studied in *D. melanogaster* using microarrays [54], but large-scale analyses in wasps are currently lacking. The genetic information provided here may help to fill this gap.

The second objective was to detect differentially-represented functions in response to symbiosis. Direct analysis of the libraries was limited by the sequencing depth at the gene level, and thus required an analysis based on the GO term level. Several genes associated with candidate functions were extracted from the current ESTs dataset, and were thoroughly studied through qRT-PCR. The current transcriptomic map can now be used as a backbone for high-throughput sequencing (*e.g.* Illumina) to provide an accurate global analysis of genes that are differentially expressed in response to symbiosis.

Through different approaches, we identified various biological processes that were transcriptionally affected by *Wolbachia* removal. Indeed, almost all the genes we studied using qRT-PCR were differently regulated in male and/or females at least in one population. The difference in gene expression was generally less than 2-fold, and could not have been detected by microarray analyses. The influence of *Wolbachia* removal on gene expression was expected in the ovaries, where the absence of *Wolbachia* dramatically alters the ovarian structure. In males however, the current absence of extended phenotype in response to *Wolbachia* removal would not have suggested changes in gene expression.

As has already been pointed out, these results must be treated with caution. In aposymbiotic individuals, antibiotic treatment could indeed have directly influenced mitochondrial metabolism [55] and gene expression because of its general cytotoxic effect. Antibiotics could also have indirectly influenced gene expression through the elimination of other bacteria (*e.g.* present in the gut community [56]). We are confident that the variations observed must have been due (or at least largely due) to *Wolbachia* infection. Indeed, we would expect the direct effects of antibiotics to affect both strains similarly. However, we found that (1) direct effects of the antibiotic treatment may be very limited, as very few genes were differentially regulated in NA males, (2) no gene (except *Transferrin*) was differentially expressed in all comparisons, and (3) as expected, the Pi3 strain was more sensitive to *Wolbachia* removal than the NA strain. These results suggest either that changes in gene expression are due to the host genotype in response to *Wolbachia* removal, or that the potential antibiotic effect impacts the expression of genes also involved in the ovarian phenotype.

As variation in dependence phenotype is determined by the host nuclear genotype [8], we studied transcriptional response to symbiosis in two populations with extreme ovarian phenotypes. However, the comparison between Pi3 and NA populations could have been obscured by their different evolutionary histories and symbiotic status regarding *Wolbachia* strains and other bacteria. To discard this hypothesis, we subsequently measured the expression of some genes in two strains originating from a same population (Saintte Foy-lès-Lyon, France), but exhibiting different ovarian phenotypes [8]. These strains were genetically related and both triply-infected, and similar patterns were observed as in the comparison between Pi3 and NA ovaries [8]. Hence, variation in gene expression in response to symbiosis must be driven by the genetic background associated with the dependence phenotype.

Growing evidence shows that the presence of a symbiont can dramatically affect host immunity [57]. For instance, *Wigglesworthia* reduces susceptibility of the tsetse fly to infection by *Trypanosoma* by modulating *PGRP-LB *[58,59], and the male-killer *Spiroplasma* weakens antimicrobial expression in *D. melanogaster *[60]. Immuno-modulation by a symbiont could thus be a way of circumventing the host’s immune system and/or to increase host fitness and ability to cope with common pathogens, thus ensuring that the symbiont is maintained within the host. Although *Wolbachia* is hidden in a host-derived vacuole, the transcriptomic analyses presented here suggest that the host organism detects its presence, and that *Wolbachia* may not only adopt an ‘immune-escape’ strategy. Indeed, *Wolbachia* seems to influence host immune system in its broad sense, including both canonical pathways and the stress response to external stimuli. These observations led us to wonder how *Wolbachia* is detected within the cell, how *Wolbachia* evades the host immune system, and what are the consequences of these manipulations on host cell physiology.

In the present study, most of the canonical immune PGRP receptors were differentially-regulated in the presence of *Wolbachia*, probably through lipoprotein or polysaccharide binding, and the outcome of the interaction tended towards under-expression of immune effectors of the Toll, Imd and JAK-STAT pathways. Even when the regulation cascade was too complex to analyze, the expression patterns of most immune genes were modified in response to symbiosis, suggesting that *Wolbachia* may adopt an active strategy of immune evasion in *A. tabida*. However, as few immune genes from the Toll signaling pathway are also known to play a role in development, expression data have to be interpreted with caution with respect to the important development defect of ovaries in aposymbiotic females. The regulation appeared to be tissue or sex-specific, immune genes being expressed to a greater extent in males than in ovarian tissues. *Wolbachia* is mainly concentrated in the ovaries of females, whereas they are spread more widely throughout the male body [61]. Hence, modulation of immune pathways could be both gene- and tissue-specific, as shown in the differential immune regulation of bacteriocytes *vs.* whole body in *Sitophilus zeamais *[62]. The immune response to *Wolbachia* also seems to be host strain-specific, with the Pi3 strain generally exhibiting a more pronounced pattern than the NA strain. Finally, the immune response to *Wolbachia* seems to be host-specific, as *Drosophila simulans* did not repress or induce antimicrobial peptides production [63], whereas the *D. melanogaster* cell line over-expressed antimicrobial peptides in response to *Wolbachia* infection [23]. Similarly, the presence of *Wolbachia* tends to increase immune gene expression in the mosquito hosts when stably introduced [20,21,50].

By comparing aposymbiotic and symbiotic tissues of *A. tabida*, we also highlighted the influence of *Wolbachia* on host immunity in its broad sense, and especially on the regulation of cell homeostasis and the oxidative environment, which are known to play a key role in physiological responses to invasion by pathogens. Indeed, processes involved in the control of the oxidative environment were highlighted both in *in silico* and *in vitro* subtractions, and confirmed by qRT-PCR. Given these observations, we further demonstrated the influence of *Wolbachia* on iron homeostasis and oxidative stress regulation in *A. tabida *[8,14]. We confirmed the differential expression of *Ferritin*, a protein involved in iron storage and transport, in males, females and ovaries from the Pi strain [14]. Since the control of iron homeostasis pleiotropically affects apoptotic and oogenetic processes, its perturbation may have played a role in the evolution of dependence [14]. Interestingly, ROS also interfere with oogenesis in mosquitoes [64] and *Drosophila *[65], probably by controlling apoptotic checkpoints [10]. The influence of *Wolbachia* on iron homeostasis was not restricted to *A. tabida*, since we demonstrated a similar effect in *D. simulans* and in an *A. aegypti* cell line [14]. Hence, processes highlighted in an association in which *Wolbachia* induces an extreme phenotype also shed light on more general processes in host/*Wolbachia* interactions. In the present study, the stress response was not restricted to iron regulation, as other chaperones and enzymes involved in detoxification were also differentially expressed in response to *Wolbachia* symbiosis, in both males and females. These results suggest a general regulation of the oxidative environment, not solely restricted to the ovaries where the phenotype is observed. Genes involved in the stress response were generally over-expressed in aposymbiotic individuals, suggesting either that *Wolbachia* has a protective effect on host physiology/immunity or that host compensatory mechanisms have been developed to reduce the harmful impact of the presence of *Wolbachia *[8]. Interestingly, we observed a differential response in Pi3 *vs.* NA strains through quantitative RT-PCR, which was confirmed in another population with similar phenotypes [8]. These results suggest that host gene expression has evolved to tolerate the presence of *Wolbachia*, and that the Pi3 genotype is more sensitive to its presence.

Finally, some striking similarities emerge when these results are compared with two other models that have been used in similar studies, but which have radically different extended phenotypes and types of relationships (*i.e. Armadillidium vulgare/Wolbachia* and *Sitophilus orizae/SOPE*) [66,67]. Functions such as oxidative stress regulation [8,14] and classical immune pathways [62] have already been highlighted, and appear again as being shared between symbiotic associations. Apoptosis has previously been highlighted in *A. tabida*, owing to the strong cellular phenotype induced by the removal of *Wolbachia *[9], but also appears to be shared by the other associations. Finally, new functions, such as autophagy, have been detected in all three associations, raising the possibility that this pathway also plays a central role in symbiotic interactions. All these functions are also shared in host-pathogen interactions, suggesting the existence of a common language between bacteria and their hosts, whatever the form their interaction takes. However, a detailed analysis of these pathways revealed that they may be under- or over-regulated, depending on the symbiotic association. These differences in gene regulation may reflect different co-evolutionary dynamics (*e.g.* an arms race or cooperation between the partners), and/or different selective pressures due to symbiont location. When symbionts are not restricted to specific tissues, deleterious side effects on other traits, *e.g.* the response to pathogens or developmental processes modulated by the pleiotropic action of genes, may indeed limit or shape the expression of these pathways.

## Conclusions

In this study, we identified 12,511 unigenes from the parasitoid wasp *A. tabida*, which can now facilitate future genetic studies on host/*Wolbachia* and host/parasitoid interactions. We also highlighted that *Wolbachia* might interfere with the expression of genes involved in development, PCD and immunity, especially through the regulation of oxidative stress. These results confirm that *Wolbachia* does not only impact its host reproduction, but may also influence more globally the biology and physiology of its hosts with potential unprecedented effects on the evolution of their life history.

## Authors' contributions

NK was involved in designing the experiments, prepared the libraries, carried out the quantitative PCR analysis, participated in the sequence analysis and drafted the manuscript. DC was involved in designing the experiments, carried out the EST data processing and analysis, and helped with the statistical analysis of expression data. HH was involved in the design and supervision of the molecular studies. FG and PW sequenced the libraries. PM was involved in designing the experiments. FV conceived and coordinated the study, was involved in its design, and helped to draft the manuscript. All the authors have read and approved the final manuscript.

## Competing interests

The authors declare that they have no competing interests.

## Supplementary Material

Additional file 1 **Primers used for quantitative RT-PCR**. Click here for file

Additional file 2 **Functions under-represented in wasp ovaries in response to *Wolbachia* infection, biological process level 6.** GO terms differentially-represented in libraries from aposymbiotic (A) and symbiotic (S) ovaries (Pi3 strain). The proportion of ESTs related to each GO function is indicated in the OA library (OA1 and OA2) and in the reference library (OS). Biological processes (level 6) are sorted relative to their A/S ratio, representing the enrichment percentage in the OA library compared to the OS library. An asterisk indicates functions shared by OA1 and OA2. Click here for file

Additional file 3 **Expression profiles of genes studied in quantitative RT-PCR** Quantitative RT-PCR was performed from symbiotic (gray) or aposymbiotic (white) extracts. The Pi3 strain exhibits a strong ovarian phenotype after *Wolbachia* removal (no eggs in the ovaries), while the NA strain produces a few eggs that do not develop normally. RNA was extracted either from 10 males or from 10 ovaries (whole ovaries for the NA strain and distal part of the ovaries for the Pi3 strain). Expression of each candidate gene was normalized by the geometric mean of three housekeeping genes. The mean of 5 biological replicates (+/- SE) is shown on the graph. *: conditions that are significantly different (Wilcoxon’s test on expression data, p-values adjusted using FDR’s correction, p-value < 0.05). Click here for file
