# Supplementary material for: Influence of Wolbachia on host gene expression in an obligatory symbiosis
Source: BMC Microbiol. 2012 Jan 18;12(Suppl 1):S7. doi: 10.1186/1471-2180-12-S1-S7 (PMC3287518; doi:10.1186/1471-2180-12-S1-S7)

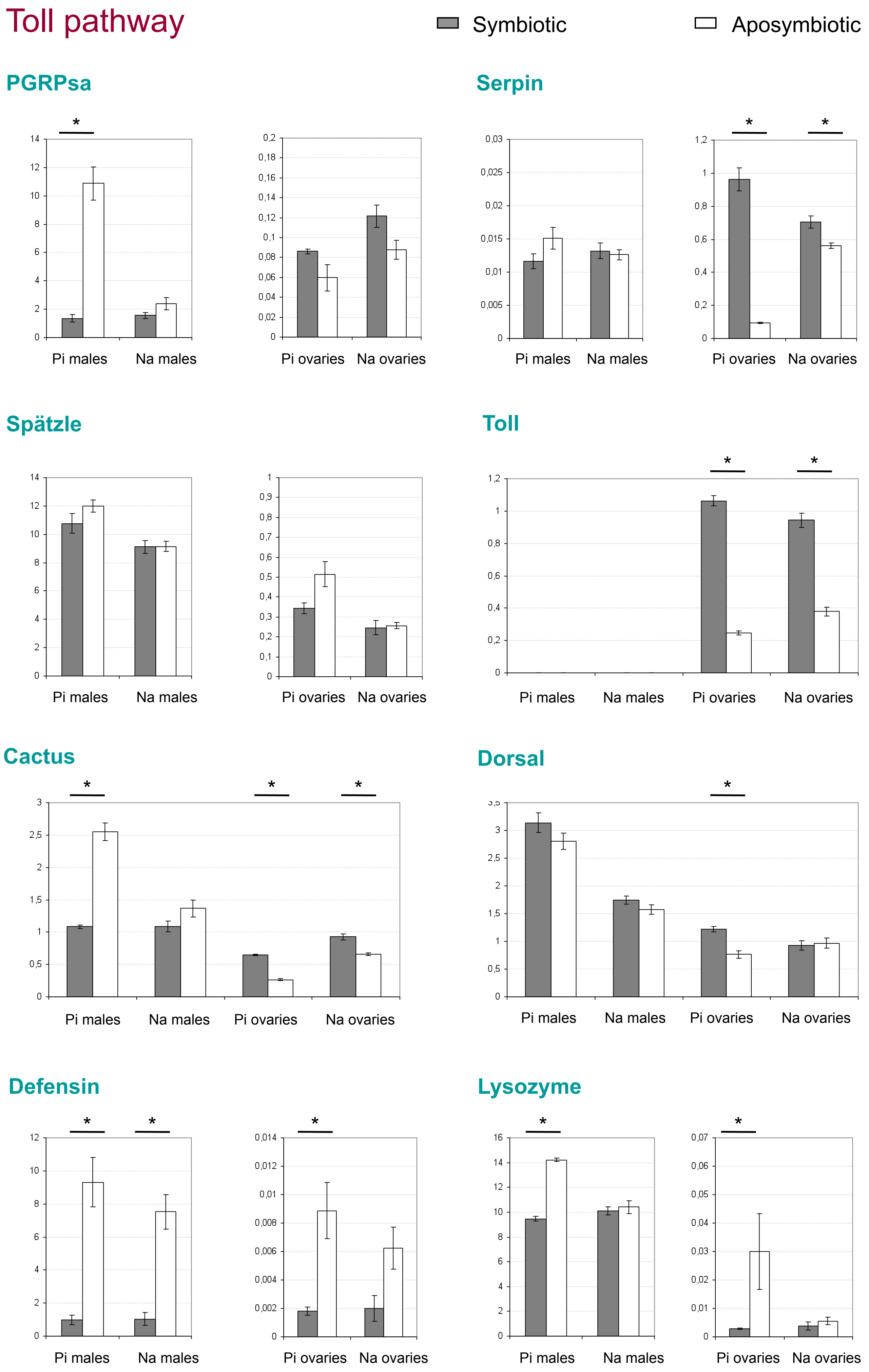
Toll pathway
Symbiotic
Aposymbiotic
PGRPsa
Pi males
Na males
Pi ovaries
Na ovaries
Serpin
Pi males
Na males
Pi ovaries
Na ovaries
Spätzle
Pi males
Na males
Pi ovaries
Na ovaries
Toll
Pi males
Na males
Pi ovaries
Na ovaries
Cactus
Pi males
Na males
Pi ovaries
Na ovaries
Dorsal
Pi males
Na males
Pi ovaries
Na ovaries
Defensin
Pi males
Na males
Pi ovaries
Na ovaries
Lysozyme
Pi males
Na males
Pi ovaries
Na ovaries

Imd pathway

Symbiotic Aposymbiotic

PGRPlc

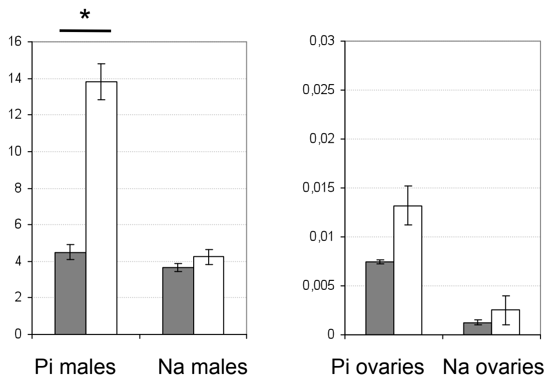

IAP *

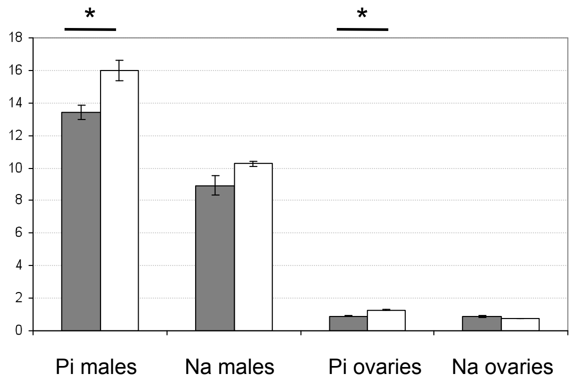

Hymenoptaecin

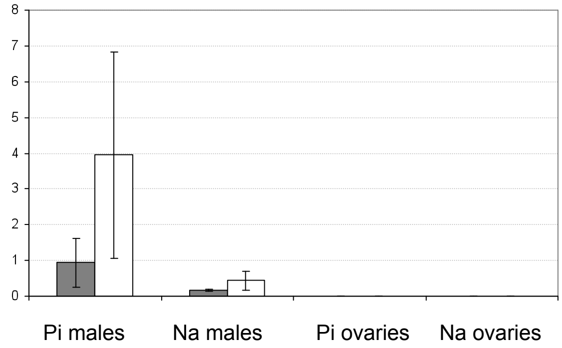

JNK pathway

MAPK / TAK1 ?

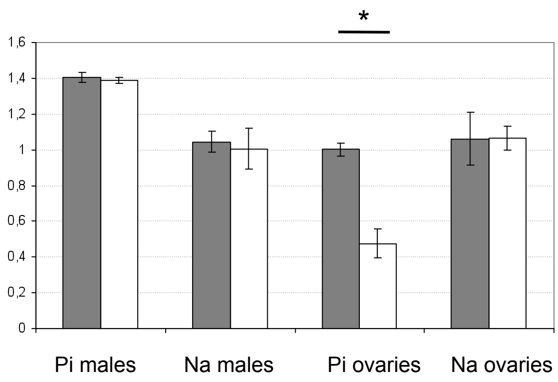

Basket

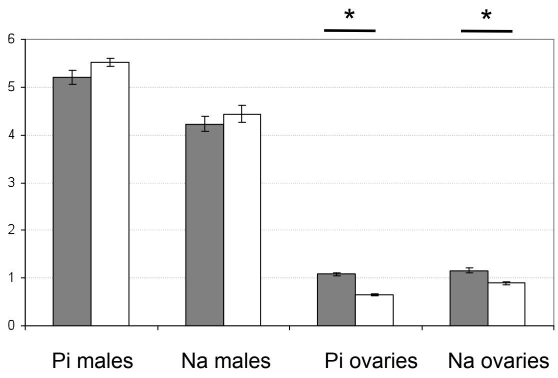

JAK-STAT pathway

Symbiotic   Aposymbiotic

Hopscotch

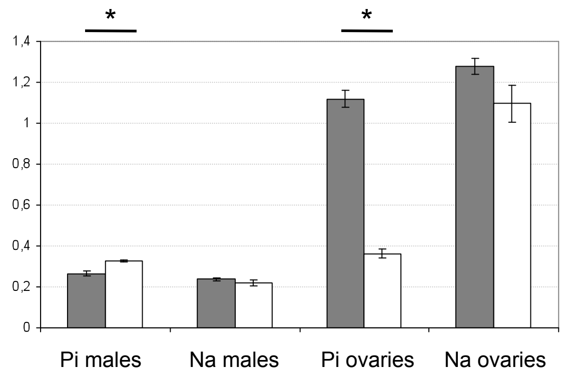

TEP

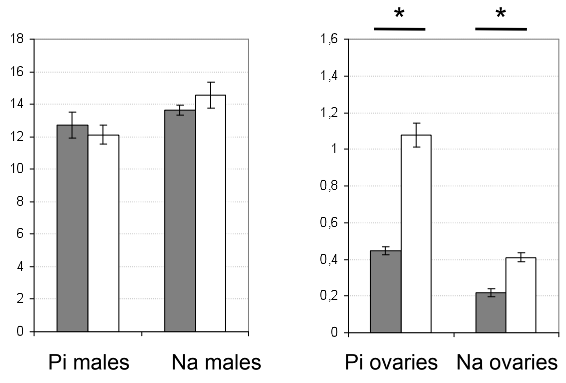

TEP2

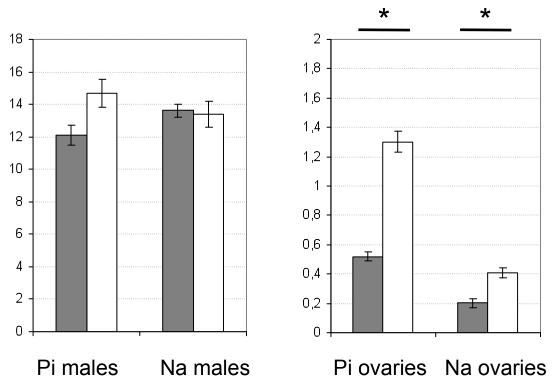

RNAi

Aubergine

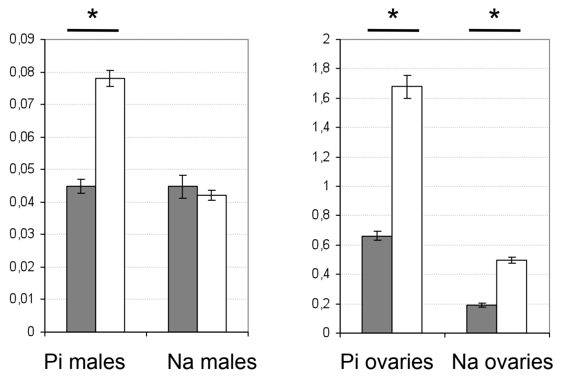

Argonaute

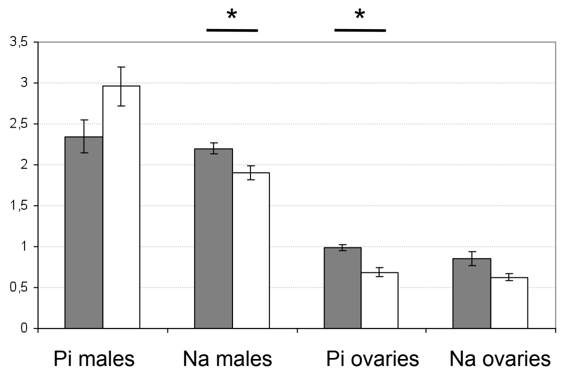

Vasa *

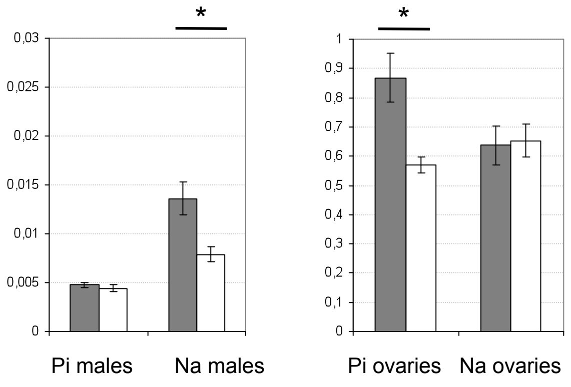

MAPK / Licorne? *

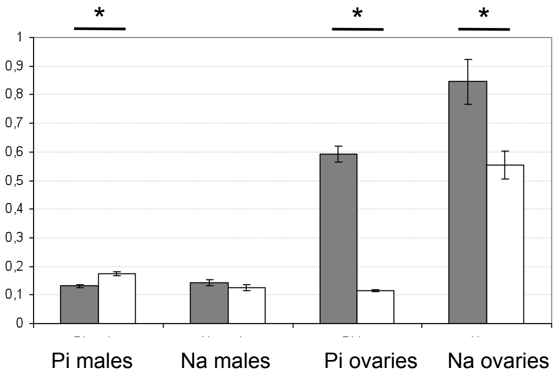

Stress

Symbiotic Aposymbiotic

Transferrin

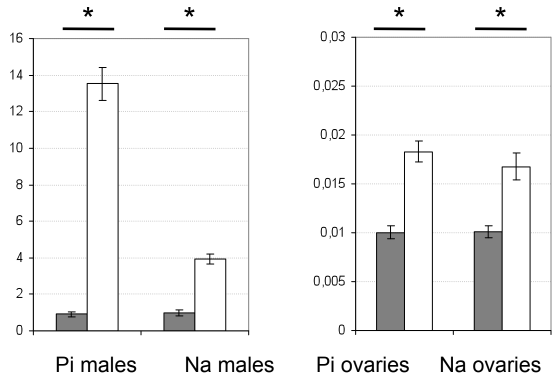

Ferritin Light Chain

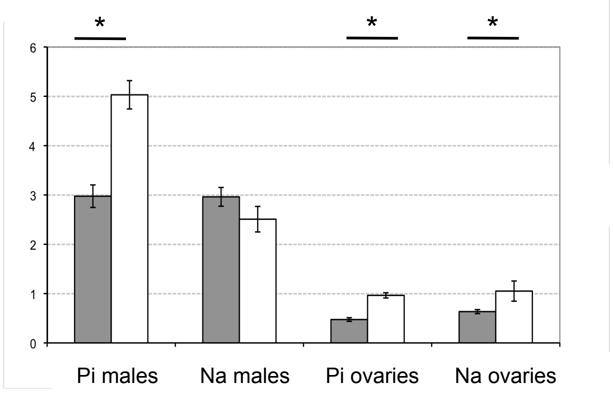

Ferritin Heavy Chain

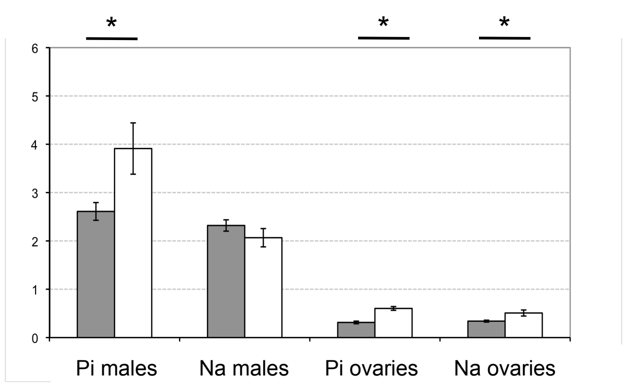

GST : Glutathione-S-transferase

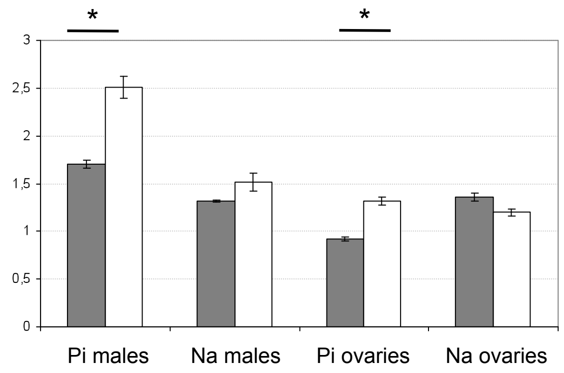

SOD : Superoxide dismutase

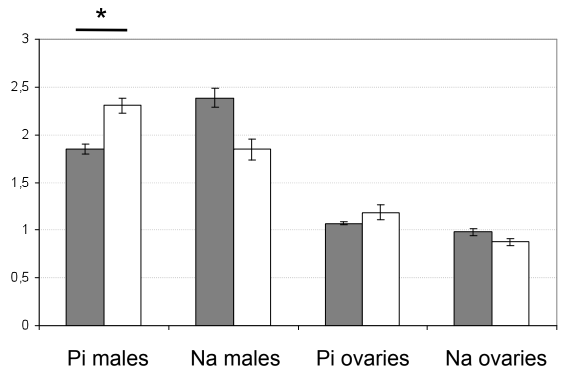

HSP70 / BIP?

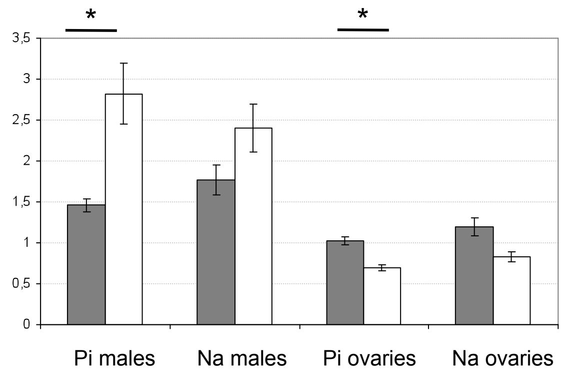

Cyclophilin

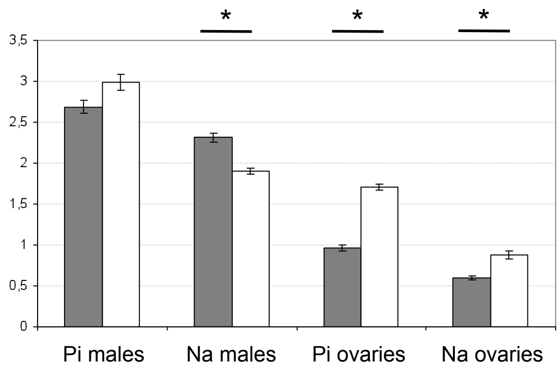

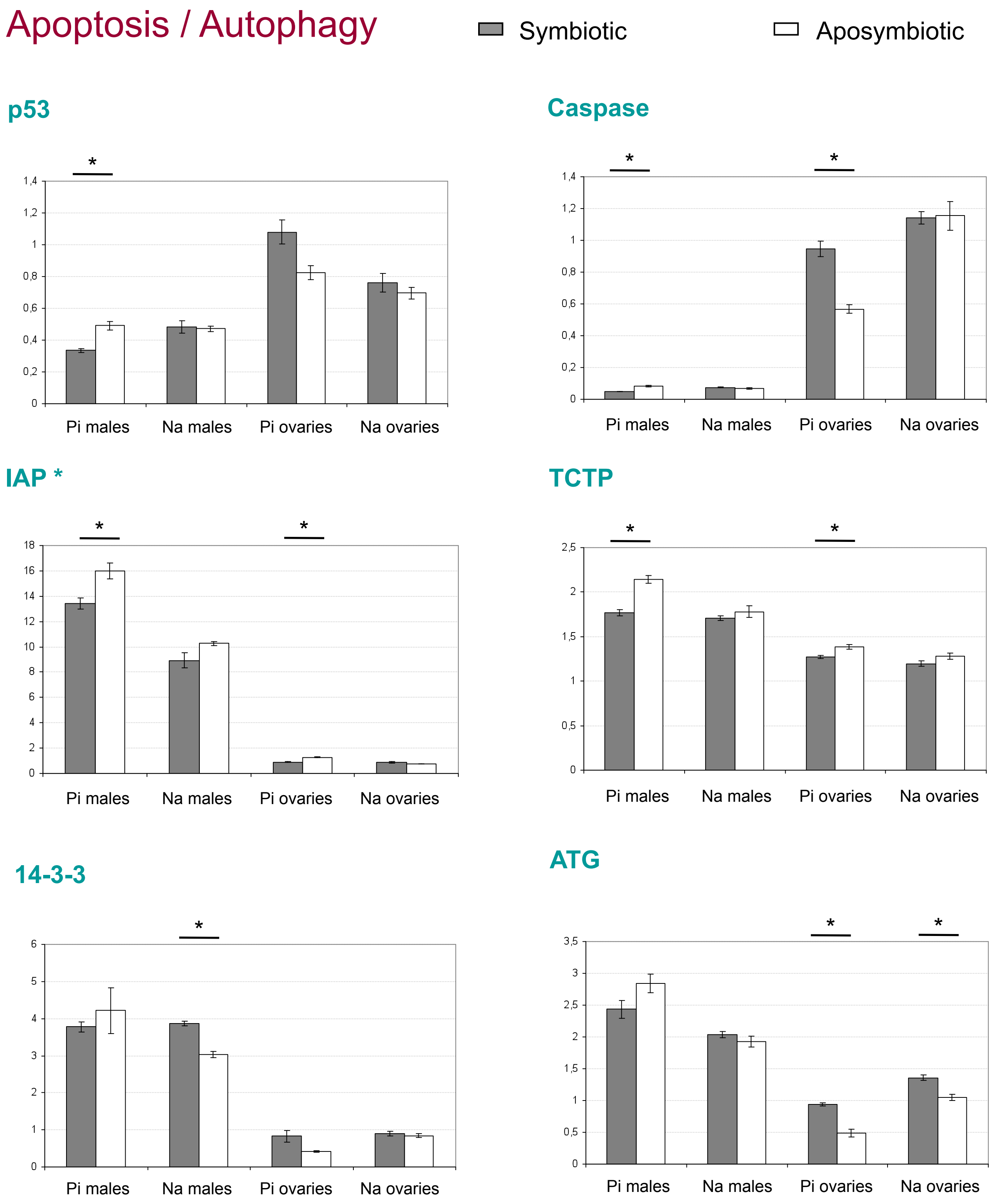
Apoptosis / Autophagy
Symbiotic
Aposymbiotic
p53
*
1,4
1,2
1
0,8
0,6
0,4
0,2
0
Pi males
Na males
Pi ovaries
Na ovaries
Caspase
*
*
1,4
1,2
1
0,8
0,6
0,4
0,2
0
Pi males
Na males
Pi ovaries
Na ovaries
IAP *
*
*
18
16
14
12
10
8
6
4
2
0
Pi males
Na males
Pi ovaries
Na ovaries
TCTP
*
*
2,5
2
1,5
1
0,5
0
Pi males
Na males
Pi ovaries
Na ovaries
14-3-3
*
6
5
4
3
2
1
0
Pi males
Na males
Pi ovaries
Na ovaries
ATG
*
*
3,5
3
2,5
2
1,5
1
0,5
0
Pi males
Na males
Pi ovaries
Na ovaries

Oogenesis

Symbiotic Aposymbioic

Sxl : Sex-lethal

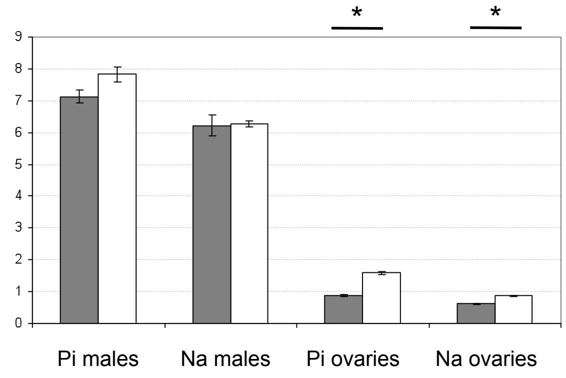

Ix : Intersex

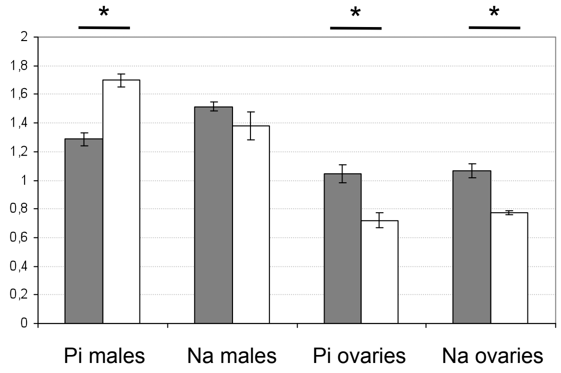

Hormone receptor-like

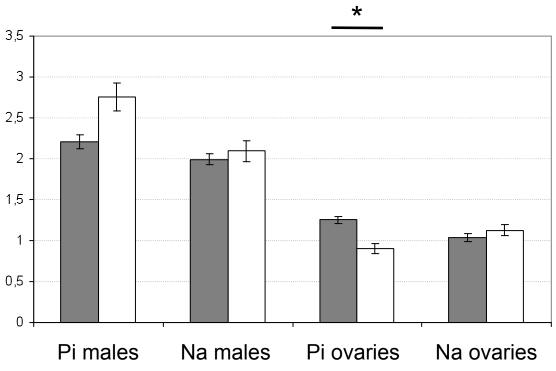

Oskar

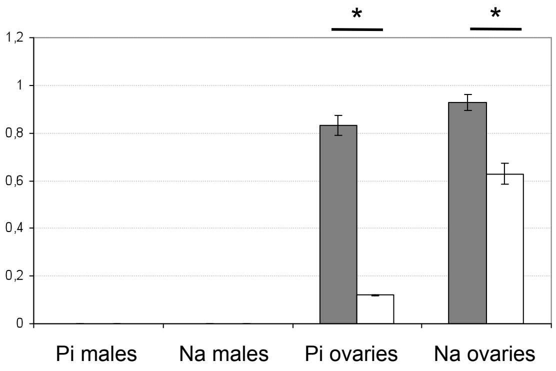

Vasa *

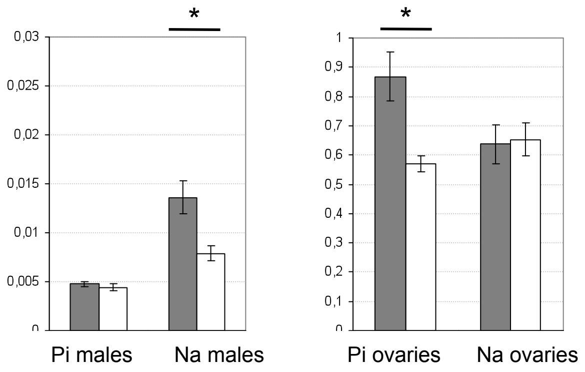

MAPK / Licorne? *

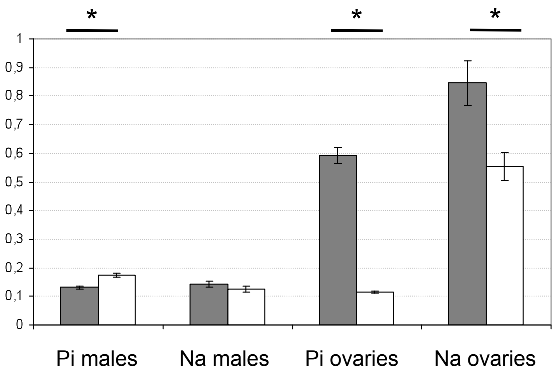

Supplement: Additional file 3 — Expression profiles of genes studied in quantitative RT-PCR Quantitative RT-PCR was performed from symbiotic (gray) or aposymbiotic (white) extracts. The Pi3 strain exhibits a strong ovarian phenotype after Wolbachia removal (no eggs in the ovaries), while the NA strain produces a few eggs that do not develop normally. RNA was extracted either from 10 males or from 10 ovaries (whole ovaries for the NA strain and distal part of the ovaries for the Pi3 strain). Expression of each candidate gene was normalized by the geometric mean of three housekeeping genes. The mean of 5 biological replicates (+/- SE) is shown on the graph. *: conditions that are significantly different (Wilcoxon’s test on expression data, p-values adjusted using FDR’s correction, p-value < 0.05). [file 1471-2180-12-S1-S7-S3.pdf]
